# *PROM1* and *CTGF* Expression in Childhood *MLL*-Rearrangement Acute Lymphoblastic Leukemia

**DOI:** 10.1155/2022/5896022

**Published:** 2022-10-14

**Authors:** Lu-lu Wang, Xue Tang, Guichi Zhou, Shilin Liu, Ying Wang, Fen Chen, Tonghui Li, Feiqiu Wen, Sixi Liu, Huirong Mai

**Affiliations:** Department of Hematology and Oncology, Shenzhen Children's Hospital, Shenzhen 518038, China

## Abstract

The prognosis of over 90% of infant acute lymphoblastic leukemia (ALL) remains poor because of harboring the mixed-lineage leukemia gene (*MLL*) fusion. To give insight into the critical coexpressed genes related to the *MLL*-rearrangement (*MLL*-R) gene in childhood acute lymphoblastic leukemia, we integrated different bioinformatic methods. First, the gene expression data of *MLL*-R ALL and normal samples from GSE13159 and GSE13164 were analyzed using “compare” function in the Oncomine database. The top 150 overexpressed and 150 underexpressed genes were identified by the Oncomine website. Then, we employed the Search Tool for the Retrieval of Interacting Genes/Proteins (STRING) to define functional genes for the 300 DEGs. The Cytoscape identified two important networks for overexpressed genes, including 35 functional genes, among which *PROM1*, *FLT3*, *CTGF*, *LGALS1*, *IGFBP7*, *ZNRF1*, and *RUNX2* were considered as the key genes because of their high expression in *MLL*-R ALL compared to the expression in other subclassification of leukemia in the MILE dataset. Further analysis of GSE68720, GSE19475, and Therapeutically Applicable Research to Generate Effective Treatments (TARGET) ALL (phase I) database confirmed the robust expression of 7 key genes in *MLL*-R compared to MLL-germline (*MLL*-G) childhood ALL. Kaplan-Meier analysis indicated that childhood ALL patients with high *PROM1* and *CTGF* expression had significantly poor overall survival. These findings suggest that *PROM1* and *CTGF* represent two potential therapeutic targets for childhood *MLL*-R ALL.

## 1. Introduction

Acute lymphoblastic leukemia (ALL) is the most common form of childhood malignancies. It is a heterogeneous hematologic disease characterized by clonal proliferation of immature lymphoid progenitor cells both in bone marrow and extramedullary sites [1]. Thanks to the development of risk-directed chemotherapy and targeted therapy against the gene mutations/fusion, the 5-year survival rate of ALL exceeds 90% [2, 3]. However, the prognosis of over 90% of infant ALL and 35–50% of childhood acute myeloid leukemia remains poor because of harboring the mixed-lineage leukemia gene (*MLL*) fusion [4–8]. For infant *MLL-*rearrangement (*MLL*-R) ALL, the 5-year event-free survival is extremely low, ranging from 20 to 40% [6]. *MLL-*R ALL has unique clinical and biologic features, including the pro-B phenotype, prenatal origin, rapid onset, early relapse, and hyperleukocytosis.

The MLL gene located in chromosome 11q23 fuses to generate chimeric genes with over 80 partners at the C-terminus and forms 135 different MLL rearrangements, of which the most common ones are AF4, AF9, AF17, ELL, and ENL [9]. These fusions are responsible for the gene expression alternation on histone methylation and transcriptional elongation. *MLL-*R activates target genes via H3K79 methylation by DOT1L, stimulation of elongation through P-TEFb, and suppression of the polycomb function [10]. However, as the breakthrough of genome-wide sequencing, a group of MLL target genes was distinguished. It has been reported that MLL fusion genes act as a global regulator by targeting more than 5000 genomic elements [11]. By far, the association between coexpressed genes and the MLL fusion gene has not been comprehensively investigated.

To better understand the whole-genome alteration of leukemia, a retrospective study named Microarray Innovations in LEukemia (MILE) was carried out in 11 laboratories across three continents and included 3334 patients with leukemia [12, 13]. Blood or bone marrow samples of acute and chronic leukemia patients were hybridized to the microarray analysis. On the Gene Expression Omnibus (GEO) website, the MILE study fell into two stages, GSE13159 and GSE13164. In this study, we explored the GSE13159 and GSE13164 datasets on the Oncomine website and defined the top 300 differentiated expressed genes (DEGs) of *MLL*-R pro-B ALL vs. normal samples. Then, we performed Gene Ontology (GO) and Kyoto Encyclopedia of Genes and Genomes (KEGG) pathway enrichment analysis for selected DEGs. Moreover, we investigated their protein-protein interaction (PPI) network based on the STRING website and selected functional genes by using Cytoscape software. The 7 key gene expression pattern and their relationship with clinical traits were searched on BloodSpot, and the UCSC Xena website was also constructed. Finally, two GEO datasets, including GSE68720 and GSE19475, studying the infant MLL-R and *MLL*-germline (*MLL-*G) ALL were employed to confirm the key genes. Exploring new genes and pathways associated with *MLL-*R ALL may help to identify potential molecular mechanisms, diagnostic markers, and therapeutic targets for *MLL-*R ALL.

## 2. Materials and Methods

### 2.1. Oncomine Analysis

Oncomine is an integrated data-mining platform that analyzes previously published or open-access cancer microarray data. Using the keywords “acute lymphoblastic leukemia” and “Cancer vs. Normal Analysis,” two studies were identified in the Oncomine database (https://www.oncomine.org) with the ID GSE13159 and GSE13164. Gene expression in pro-B ALL vs. normal was analyzed by the “compare” function in the Oncomine database.

According to the description of MILE, all of the pro-B ALL patients harbored MLL fusion in GSE13159 and GSE13164. The result orders genes by median rank across the two analyses and displays the corresponding *p* values. The overexpressed and underexpressed genes with rank orders above 150 and *p* < 0.05 were selected for further analysis.

### 2.2. GO and KEGG Enrichment Analyses

The top 150 over- and underexpressed genes were taken into DAVID website separately, analyzed by GO and KEGG enrichment (*p* < 0.05).

### 2.3. Protein-Protein Interaction Network

The 300 DEGs were taken into Search Tool for the Retrieval of Interacting Genes/Proteins (STRING) with the maximum number of interactors = 0 and a confidence score ≥ 0.4 as the cutoff criteria. Then, to understand the function of the overexpressed gene, the biofunctional modules in the top 150 overexpressed genes were explored using a plug-in MCODE in Cytoscape with a node score cutoff of 0.2, degree cutoff of 2, and *k*-Core of 2. The top two gene modules with the highest MCODE scores were selected from the network. Then, the genes were taken into DAVID, as demonstrated above. KEGG enrichment analyses were carried out with the significance threshold *p* < 0.05.

### 2.4. BloodSpot Website Analysis

BloodSpot is a database of mRNA expression in healthy and malignant hematopoiesis and includes data from both humans and mice [14]. The functional gene names were input into the search bar as a query. Gene expression data of the MILE study were identified on the BloodSpot website.

### 2.5. UCSC Xena Analysis

The gene expression, MLL status, and minimal residual disease (MRD) monitor were verified and analyzed in TARGET ALL (phase I) using the UCSC Xena browser.

### 2.6. Data Collection and Gene Expression Analysis in the GEO Dataset

Microarray expression data of GSE68720 and GSE19475 were downloaded from the GEO database. To explore the relationship between infant *MLL-*R ALL and *MLL-*G ALL, cel files of 17 *MLL-*G ALL samples and 80 *MLL-*R ALL samples from GSE68720 and 14 *MLL-*G ALL and 58 *MLL-*R ALL samples from GSE19475 were selected. The robust multiarray average in R was applied to explore the gene expression data in the cel files, including background correction, normalization, and summarization. All of the above operations were run with scripts in the R 3.6.3 version. The ggplot2 package in R was used to show the heat map of key genes.

### 2.7. Kaplan-Meier Analysis

Gene expression was obtained from UCSC Xena website, and the clinical survival information of TARGET ALL (phase I) was downloaded from the official TARGET database website. The ggplot2 of R software was used to plot the Kaplan-Meier survival curve. The TARGET ALL (phase I) project is obtained from patients enrolled on biology studies and clinical trials managed through the COG, POG 9906 (clinical trial for patients with newly diagnosed ALL between March 2000 and April 2003 that were defined as high risk for relapse). Patient samples for full characterization were chosen based on the following criteria: the disease onset at >9 years of age, did not have white blood cell count > 50000/*μ*L, did not express the BCR/ABL fusion gene, were not known to be hypodiploid (DNA index > 0.95), and achieved remission (fewer than 5% blasts) following the standard two rounds of induction therapy. The primary patient samples were collected at diagnosis, and gene expression was analyzed following the protocol of Human Genome U133 Plus 2.0 Array (Affymetrix).

### 2.8. Statistical Analysis

Student *t*-test of variance was used for comparing the statistical differences of gene expression of samples in GSE19475 and GSE68720. All the analyzes were two sided and *p* < 0.05 was considered to be significant.

## 3. Results

### 3.1. Identification of the Top DEGs in *MLL-*r ALL

**Figure 1:**
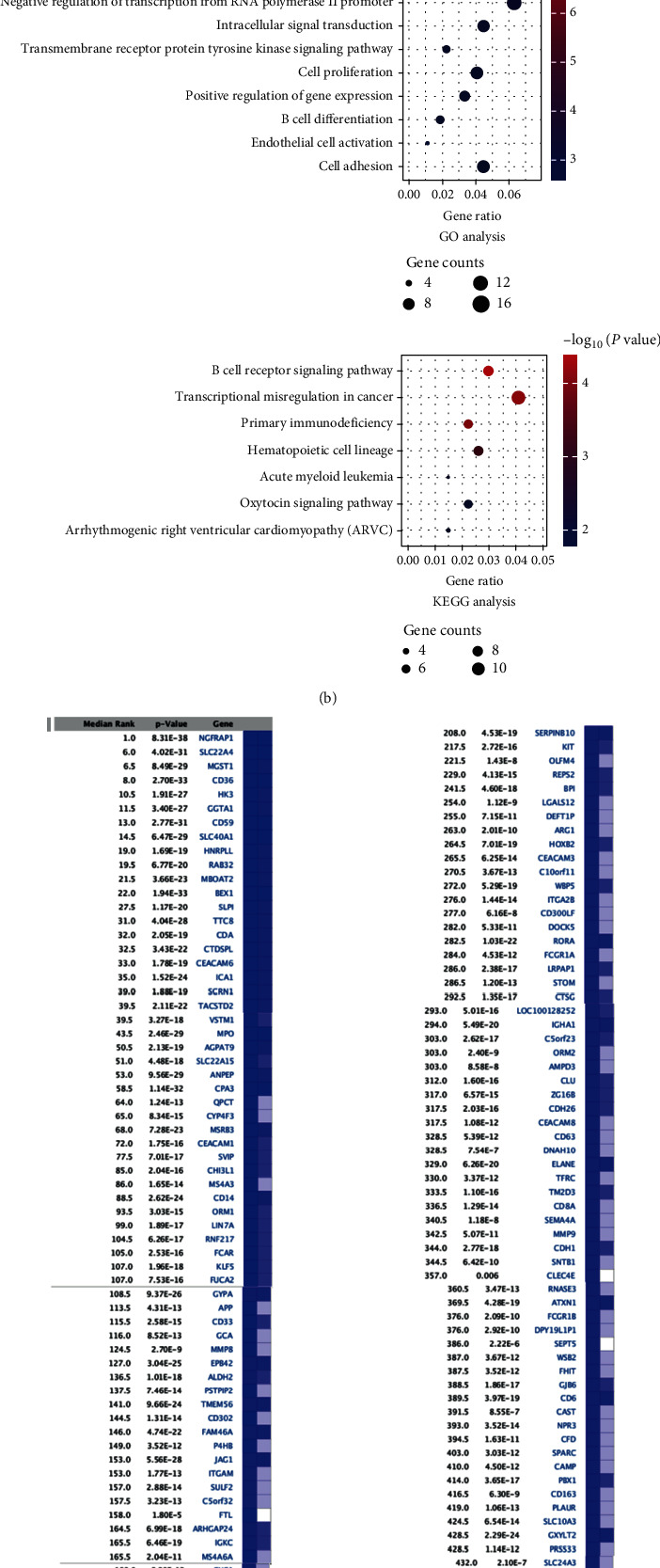
The expression profiles of *MLL*-R pro-B ALL in the MILE study. (a) Top 150 overexpressed DEGs were shown in the table. (b) GO and KEGG enrichment analyses for top 150 overexpressed DEGs. (c) Top 150 underexpressed DEGs were shown in the table. (d) GO and KEGG enrichment analyses for top 150 underexpressed DEGs.

The gene expression data of *MLL-*R ALL and normal samples from GSE13159 and GSE13164 were analyzed using the “compare” function in the Oncomine database. The median rank of the overexpressed and underexpressed genes with rank orders above 150 was identified as the genes and selected for further analyses (Figures 1(a) and 1(c)).

Based on the result from the DAVID online analysis tool, the KEGG pathway and GO analysis were carried out to better understand the biological function of the key DEGs in *MLL-*R ALL. The GO enrichment analysis result showed that the overexpressed genes were mainly enriched in biological processes, including the B cell receptor signaling pathway, B cell activation, and negative regulation of transcription from the RNA polymerase II promoter, while KEGG pathway analysis showed that the result was significantly enriched in the B cell receptor signaling pathway, transcriptional misregulation in cancer, and primary immunodeficiency (Figure 1(b)). As for underexpressed genes, GO enrichement analysis demonstrated that they were mainly enriched in platelet degranulation pathway. KEGG pathway analysis showed that the underexpressed genes were mainly enriched in the hematopoietic cell lineage pathway (Figure 1(d)).

**Figure 2:**
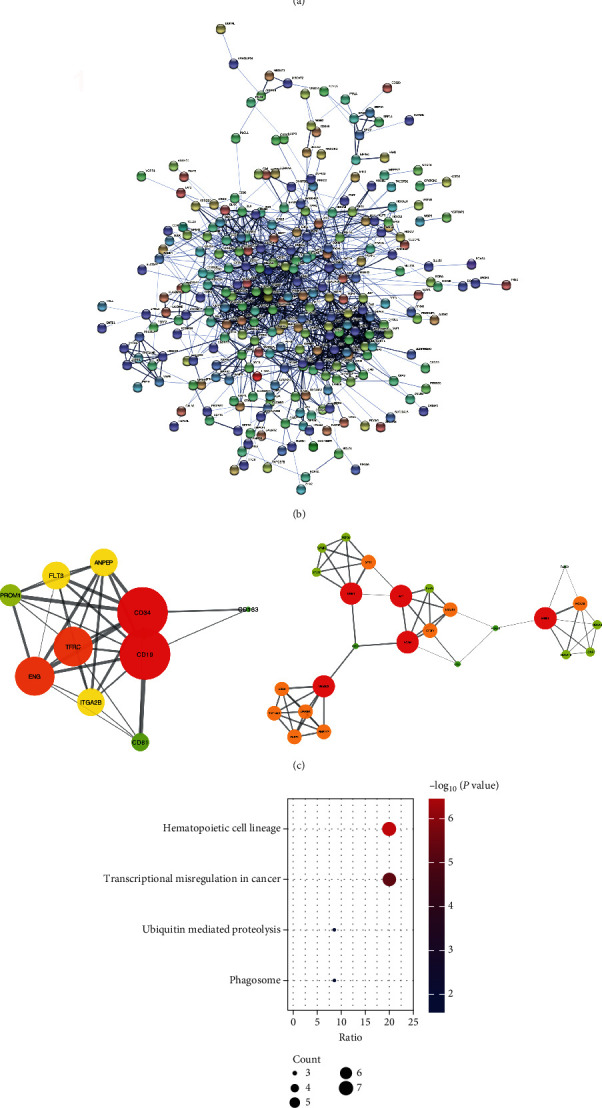
PPI network of DEGs by STRING. (a) The Venn diagram showed the top 300 DEGs. (b) The PPI network was constructed by STRING based on the top 300 DEGs. (c) The functional genes of overexpressed DEGs found by MCODE made up 2 critical subnetworks. (d) KEGG pathway analysis for functional genes.

A functional gene usually refers to what is significant in regulation and biological processes and closely interacts with other genes in a network. A total of 300 DEGs, including 150 overexpressed and 150 underexpressed genes, were shown in the overlap of the Venn diagram (Figure 2(a)). To further investigate the function of the DEGs in the GSE13159 and GSE13164 at the protein level, the STRING was employed to screen for functional genes. The PPI network consisted of 295 nodes and 1378 edges (Figure 2(b)). Afterwards, the interactive relationship of overexpressed genes was analyzed separately in Cytoscape. The MCODE, a plug-in in Cytoscape, was employed to calculate the *k*-Core of each gene. The top two significant modules in MCODE with high scores were selected from the PPI network, including module A (MCODE score = 7.556 with 10 nodes) and module B (MCODE score = 4.75 with 25 nodes) (Figure 2(c)). These genes were involved in 4 important KEGG pathways, including the hematopoietic cell lineage, transcriptional misregulation in cancer, ubiquitin-mediated proteolysis, and phagosome (Figure 2(d)).

### 3.2. Validation of Key Genes in *MLL-*R ALL

**Figure 3:**
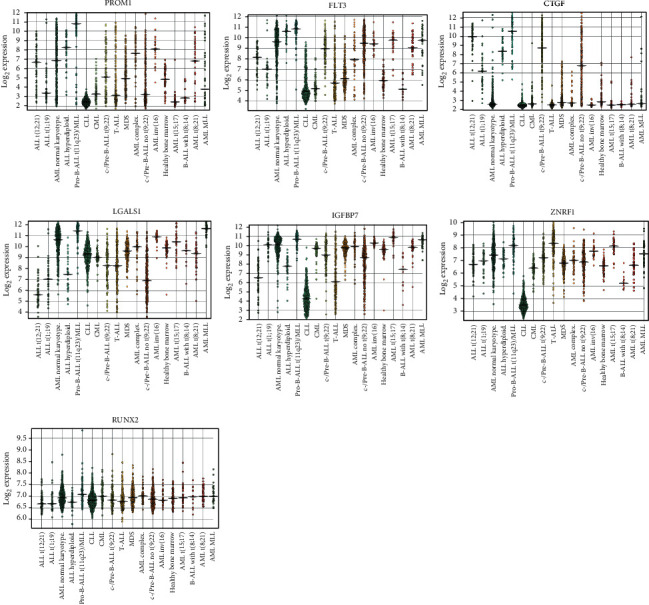
Key gene expression on the BloodSpot website. The box plot showed the expression of *PROM1*, *FLT3*, *CTGF*, *LGALS1*, *IGFBP7*, *ZNRF1*, and *RUNX2* in different subclassifications of ALL on the BloodSpot website.

To demonstrate the role of 35 functional genes in ALL subclassifications, we used the BloodSpot website to check their expression in different subclassifications of leukemia. As shown in Figure 3, *PROM1*, *FLT3*, *CTGF*, *LGALS1*, *IGFBP7*, *ZNRF1*, and *RUNX2* were found highly expressed in the *MLL*-R pro-B ALL compared to the other subclassification of leukemia.

**Figure 4:**
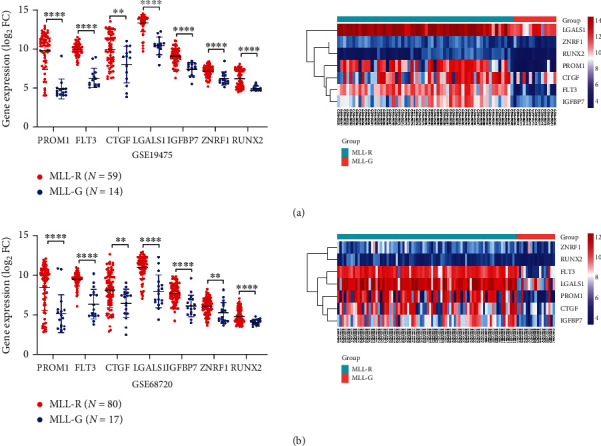
Key gene expression in *MLL-*R compared to *MLL*-G in GSE19475 and GSE68720. (a) Scatter plot and heat map of 7 key gene expression in GSE19475 according to the value of |logFC|. (b) Scatter plot and heat map of 7 key gene expression in GSE68720 according to the value of |logFC|.

**Figure 5:**
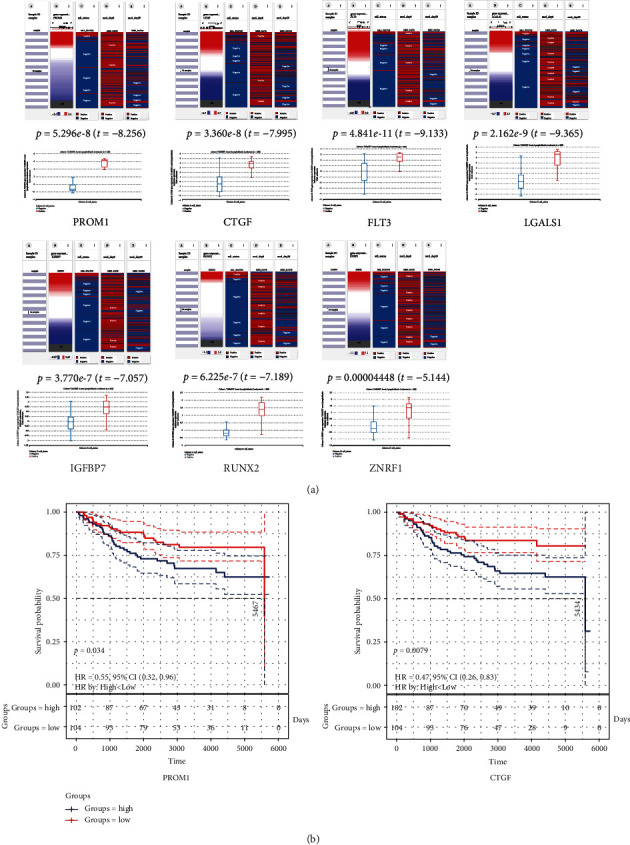
Kaplan-Meier analysis of *PROM1* and *CTGF* expression in childhood ALL. (a) The relationship of key gene expression with the MLL status in the TARGET ALL (phase I) dataset. (b) Survival cure comparing patients with high (blue) vs. low (red) *PROM1* expression was plotted using a log-rank test (HR = 0.61 (0.43–0.87), *P* = 0.034), as well as the *CTGF* (HR = 0.47 (0.26–0.83), *P* = 0.0079).

To further verify the identified 7 key genes in *MLL*-R ALL, we detected the expression of *PROM1*, *FLT3*, *CTGF*, *LGALS1*, *IGFBP7*, *ZNRF1*, and *RUNX2* between *MLL*-R ALL and *MLL*-G ALL in GSE68720 and GSE19475 datasets by using the R software. In both GSE68720 and GSE19475 datasets, the 7 key genes were significantly overexpressed in *MLL*-R compared to the *MLL-*G ALL samples, especially for *PROM1*. The heat map of the 7 key genes were shown in Figures 4(a) and 4(b). Further analysis in UCSC Xena demonstrated that high expression of these genes was significantly associated with the MLL status in the TARGET ALL (phase I) database, presenting a high correlation with the status of MLL fusion (Figure 5(a)). These results demonstrated that 7 key genes have extremely high expression in *MLL-*R ALL and maybe the critical targets for MLL fusion.

### 3.3. Survival Analysis of *PROM1* and *CTGF* in Childhood ALL

To delineate the prognostic value of potential key genes, the overall survival analyses of 7 key gene expression were detected in the TARGET ALL (phase I). The result showed that a high expression level of *PROM1* and *CTGF* was associated with inferior overall survival of ALL (Figure 5(b)).

## 4. Discussion

Although studies have demonstrated numerous fusion partner proteins, the target genes of *MLL*-fusion and the molecular mechanism involved in target genes were poorly understood. In the past decade, genomic analyses have revolutionized our understanding of the coexpression network in *MLL*-R ALL. *HOX* cluster genes and its cofactor *MEIS1* were the most well-known target genes for the *MLL* fusion gene [15]. Both *HOXA* genes and *MEIS1* are highly expressed in the stem cells and early progenitor cells. MLL drives the proliferation and self-renewal of immature hematopoietic cells by upregulating posterior *HOX* genes and their cofactor *MEIS1* [16, 17]. Coincidentally, in this study, we examined the Oncomine website and investigated DEGs related to *MLL*-R ALL in the MILE study. Using PPI analysis, the critical pathway of functional genes was found involved in the hematopoietic cell lineage and transcriptional misregulation in cancer, including *HOXA10*, *MEIS1*, *FLT3*, *CD14*, *PROM1*, *RUNX2*, and *RUNX1* (data not shown), indicating the dominant roles of *HOXA* and *MEIS1* in *MLL*-R ALL.

Posttranslational modifications of PROM1 play a critical role in *MLL*-R ALL [18, 19]. It was reported that AF4 recruited and activated DOT1L at the H3K79me2/3 locus of the *PROM1* promoter, which is required for the growth of *MLL*-AF4 B-cell ALL cells [20–22]. CD133 is a kind of transmembrane glycoprotein encoded by the *PROM1* gene. It is associated with cancer stem cells in diverse human tumors, including brain, liver, stomach, endometrium, ovary, and colorectum and gliomas and medulloblastoma [23]. Recent studies demonstrated that CD19/CD133 tandem CAR T induces robust cytotoxicity against CD19+ CD133+ and CD19− CD133+ B-cell lines, suggesting CD133 a promising target *MLL*-R ALL immunotherapy [24]. However, this study was challenged by “on-target off-tumor” myeloablative and life-threatening toxicity, because the CD133 was expressed in the hematopoietic stem and progenitor cells [25].

CTGF, CCN2 as the official name, is an extracellular matrix- (ECM) associated protein of 36–38 kDa and a member of the CCN family of proteins. It plays a great role in cell adhesion, proliferation, migration, and differentiation and improves the development of numerous tumor metastases [26–29]. Interestingly, elevated *CTGF* expression is also a feature of precursor B-cell ALL [30–33]. By analyzing COG trial P9906, high expression of *BMPR1B*, *CTGF*, *TTYH2*, *IGJ*, *NT5E (CD73)*, *CDC42EP3*, and *TSPAN7* was found to be associated with poor outcomes in precursor-B ALL patients [34]. Ruling out the possibility of structure alternation, amplification, or base mutation, Welch et al. demonstrated that the *CTGF* locus is hypomethylated in pediatric pre-B ALL [35]. Anti-CTGF monoclonal antibody attenuated tumor growth of precursor-B ALL from pediatric patients propagated in mice [36]. Here in this study, *PROM1* and *CTGF* were overexpressed in *MLL*-R compared to *MLL*-G patients and those with high *PROM1* and *CTGF* expression had significantly poor OS (Figure 5(b)). Further in vitro, in vivo, and clinical studies are warranted to delineate the role of *PROM1* and *CTGF* in *MLL*-R ALL.

In conclusion, we first demonstrated the top DEGs of GSE13159 and GSE13164 by using the Oncomine website. After integrated analyses, we identified from the 300 DEG genes that *PROM1*, *FLT3*, *CTGF*, *LGALS1*, *IGFBP7*, *ZNRF1*, and *RUNX2* were the key genes, as they were highly expressed in *MLL*-R ALL compared to *MLL*-G ALL. Further investigation demonstrated that *PROM1* and *CTGF* were the poor prognostic markers for childhood *MLL*-R ALL. Thus, we provide an insight into ALL that *PROM1* and *CTGF* may be the novel potential target genes for the MLL fusion gene in childhood *MLL*-R ALL.

## Data Availability

The data that support the findings of this study are available from the corresponding author upon reasonable request.

## Abbreviations

ALL: Acute lymphoblastic leukemia
DEG: Differentiated expressed gene
GEO: Gene Expression Omnibus
GO: Gene Ontology
HR: Hazard ratio
KEGG: Kyoto Encyclopedia of Genes and Genomes
MILE: Microarray Innovations in LEukemia
MLL: Mixed-lineage leukemia gene
MLL-G: Mixed-lineage leukemia gene-germline
MLL-R: Mixed-lineage leukemia gene-rearrangement
OS: Overall survival
PPI: Protein-protein interaction
STRING: Search Tool for the Retrieval of Interacting Genes/Proteins
TARGET: Therapeutically Applicable Research to Generate Effective Treatments.
